# Molecular-Guided Precision Oncology in Cancer of Unknown Primary: A State-of-the-Art Perspective

**DOI:** 10.3390/jpm16020080

**Published:** 2026-02-01

**Authors:** Vivek Subbiah, Elie Rassy, Frank A. Greco

**Affiliations:** 1 Sarah Cannon Research Institute, Nashville, TN 37203, USA; viveksubbiah@outlook.com; 2 Department of Medical Oncology, Gustave Roussy, University Paris-Saclay, 94805 Villejuif, France; elie.el-rassy@gustaveroussy.fr; 3 CESP, INSERM U1018, Université Paris-Saclay, 94807 Villejuif, France; 4 Sarah Cannon Cancer Center, Tennessee Oncology, Greco-Hainsworth Centers for Research, Nashville, TN 37203, USA

**Keywords:** carcinoma of unknown primary, precision oncology, molecular profiling, gene expression profiling, tumor-agnostic therapy, biomarker-driven treatment

## Abstract

Cancer of unknown primary (CUP) is evolving from a diagnosis of exclusion treated with empirical chemotherapy to a molecularly defined entity amenable to precision-based interventions. This heterogeneous entity, comprising 2–3% of all metastatic malignancies, encompasses diverse cancers with clinically occult primary sites at diagnosis after a thorough workup. Recent landmark trials including CUPISCO and Fudan CUP-001 have demonstrated significant survival improvements with molecularly guided therapies compared to empirical chemotherapy, fundamentally enhancing and complementing traditional organ-centric treatment paradigms. This review synthesizes the current evidence supporting molecular diagnostics, tumor-agnostic therapies, and precision-based approaches in CUP management. We examine the clinical utility of comprehensive genomic profiling, gene expression profiling, and liquid biopsy technologies, while addressing implementation challenges and future directions. The integration of molecular tumor boards and the emergence of tissue/tissue-of-origin agnostic therapies herald a new era where CUP transitions from therapeutic nihilism to personalized oncology. As molecular technologies advance and targeted therapies proliferate, CUP may no longer represent a diagnosis of exclusion but rather an opportunity for molecularly informed precision care.

## 1. Introduction

Cancer of unknown primary (CUP) has historically represented one of the most challenging oncologic clinical scenarios—a heterogeneous syndrome characterized by metastatic disease without an identifiable primary tumor despite comprehensive diagnostic evaluation [1]. Far from being a rare entity, CUP affects an estimated 37,350 patients annually in the United States, surpassing the incidence of multiple myeloma, chronic lymphocytic leukemia, and several organ-specific de novo metastatic cancers, including pancreatic and renal carcinomas [2]. Globally, CUP comprises 2–3% of all metastatic malignancies, underscoring its significant clinical burden.

The absence of an identified anatomical primary site has traditionally complicated staging, prognostication, and treatment selection, relegating most patients to empirical chemotherapy with limited survival benefits [3]. This organ-centric paradigm, rooted in anatomical classification systems, has yielded disappointing outcomes with median overall survival typically ranging from 6 to 16 months [3]. However, the landscape of CUP management is undergoing a fundamental transformation, driven by advances in molecular diagnostics (clinical next-generation sequencing adoption), precision oncology (availability of genomically targeted therapies and immunotherapies), and our evolving understanding of cancer biology [4,5,6].

Recent pivotal trials have demonstrated that molecularly guided therapies can significantly improve outcomes compared to empirical chemotherapy, challenging the conventional wisdom that has dominated CUP management for decades [7,8]. This paradigm shift represents more than incremental progress—it signals a fundamental reconceptualization of CUP from a diagnosis of exclusion to a molecularly defined entity amenable to precision-based interventions [6].

## 2. Historical Context and Evolution of CUP Management

### 2.1. Traditional Classification and Treatment Approaches

Historically, CUP has been dichotomized into “favorable” and “unfavorable” subtypes based on clinicopathological features [9]. Approximately 20% of cases present with characteristics suggestive of a responsive or treatable presumptive primary tumor, previously defined as favorable CUP. The most common metastatic sites in CUP are, e.g., liver, lung, bone, lymph nodes. Novel qualitative assessments such as PET-CT have improved diagnostic yield but still fail to identify primaries in a subset of patients. Site-specific therapy (SST) in these cases yields outcomes comparable to those seen in patients with known primary tumors of similar histology.

The remaining 80% of cases, lacking definitive clinicopathological features pointing to a specific primary site, were classified as unfavorable CUP. For these patients, empirical chemotherapy became the standard of care, typically employing broad-spectrum cytotoxic regimens such as carboplatin/paclitaxel or gemcitabine/cisplatin. Despite decades of clinical trials, this approach has yielded consistently disappointing results with minimal improvement in overall survival.

### 2.2. Early Molecular Approaches and Initial Setbacks

The advent of and experience with gene expression profiling (GEP) approximately 15 years ago offered the first molecular approach to CUP diagnosis and management [10]. These platforms promised to identify tissue of origin (TOO) and guide site-specific therapy based on molecular signatures rather than morphological features alone. A large, single-arm, phase II study conducted by a cooperative group demonstrated improved overall survival in patients with cancer of unknown primary (CUP) who received assay-directed, site-specific therapy. These results compare favorably with historical outcomes from empiric CUP treatment regimens, thereby emphasizing the clinical value of GEP in CUP.

However, early randomized studies evaluating GEP-guided treatment yielded disappointing results. A phase II study conducted from 2008 to 2015 and a phase III trial from 2012 to 2018 failed to demonstrate improved outcomes with SST compared to empirical chemotherapy [11]. Several factors contributed to these negative results. The limited therapeutic options available at the time meant that site-specific therapies were predominantly chemotherapy regimens similar to empirical treatments [11]. Furthermore, the absence of targeted agents was notable, as molecularly targeted therapies and immunotherapy were not yet available or integrated into these studies. Additionally, technological limitations of early GEP platforms resulted in lower accuracy and limited cancer type representation. Consequently, major clinical practice guidelines from the National Comprehensive Cancer Network (NCCN) and European Society for Medical Oncology (ESMO) did not recommend routine use of GEP, reinforcing reliance on empirical chemotherapy approaches.

## 3. The Molecular Revolution in CUP Management

### 3.1. Landmark Clinical Trials 

Landmark trials in cancer of unknown primary (CUP) have fundamentally altered the management landscape. FUDAN-CUP-01, CUPISCO, and GEFCAPI represent pivotal studies that have evaluated molecularly guided therapy, site-specific treatment approaches, and precision medicine strategies in CUP. Key features and outcomes of these major trials are outlined below. (Table 1).

#### The Fudan CUP-001 Study

The prospective phase III Fudan CUP-001 study marked a defining moment in CUP management [8]. This randomized trial enrolled 182 patients with unfavorable CUP, comparing GEP-directed SST with empirical chemotherapy [9]. The results demonstrated a statistically significant and clinically meaningful improvement in survival for patients in the GEP-directed group [14]. Critically, this study differed from earlier negative trials by incorporating targeted therapies and immunotherapy when available and indicated. However, only 26% of patients who would be expected to benefit from immunotherapy actually received these agents, suggesting that the survival benefit might have been even greater with optimal matching and better drug access.

### 3.2. The CUPISCO Trial

The CUPISCO randomized phase II trial represented the largest evaluation of molecularly guided therapy in CUP to date, involving 573 patients with non-squamous, unfavorable CUP [7]. This innovative study design required the initial comprehensive genomic profiling (CGP) of tissue or blood, followed by three cycles of empirical chemotherapy [15]. Patients without progressive disease (438 patients) were then randomized 3:1 to receive either molecularly targeted therapies (including immunotherapy) or continuation of empirical chemotherapy. The study demonstrated a significant and clinically beneficial improvement in progression-free survival for patients treated with molecularly targeted therapy versus empirical chemotherapy. This tumor-agnostic approach, guided by molecular alterations rather than presumed tissue of origin since there was no attempt to determine TOO, represented a new template toward precision oncology in CUP management [7].

### 3.3. Meta-Analysis of CUP Trials

A systematic review and meta-analysis encompassing six prospective studies (including four randomized controlled trials) with 1644 patients confirmed meaningful survival improvements with molecularly directed therapy versus empirical chemotherapy [13]. This analysis included the Fudan CUP-001 study, CUPISCO trial, and earlier negative studies, providing evidence for the practice-changing potential of molecular approaches [13]. Squamous cell carcinoma (SCC) has often been excluded from molecular CUP trials due to its distinct clinical behavior and relatively favorable prognosis when localized to cervical lymph nodes. The American Cancer Society and NCI guidelines recommend site-specific management for SCC CUPs, often involving head and neck protocols. Pembrolizumab, nivolumab and nivolumab and ipilimumab have demonstrated clinical activity, especially in MSI- and TMB-high cases. Although PD-L1 is not a perfect predictive marker, when added with MSI-H and TMB-H, it has better predictability of response.

### 3.4. Complementary Molecular Strategies

The success of both GEP-guided and CGP-guided approaches has established two complementary strategies for CUP management [16,17]. The first strategy involves GEP-directed site-specific therapy based on the predicted tissue of origin, while the second encompasses CGP-guided tumor-agnostic therapy based on actionable molecular alterations regardless of histology [4]. These approaches may be synergistic, as CGP can provide specific therapeutic guidance even within GEP-diagnosed primary tumor categories [18,19,20,21].

## 4. Molecular Diagnostic Technologies

### 4.1. Comprehensive Genomic Profiling (CGP)

Modern CGP platforms enable the identification of actionable mutations, gene fusions, microsatellite instability, tumor mutational burden, and other biomarkers relevant to targeted therapy selection [4,18,19,20,21] (Table 2). These assays can be performed on tissue specimens or circulating tumor DNA (ctDNA), providing flexibility in sample acquisition and real-time monitoring capabilities.

CGP offers several key advantages in the CUP setting [22]. The broad biomarker coverage allows for single assay evaluation of hundreds of genes, providing comprehensive molecular characterization in a single test [16,22]. The therapeutic actionability of CGP enables direct guidance for FDA-approved targeted therapies, streamlining the translation from molecular findings to clinical interventions [23,24,25]. Furthermore, the tumor-agnostic applicability of CGP allows for treatment selection independent of tissue of origin, which is particularly relevant in the CUP context where primary site identification remains challenging. Finally, the dynamic monitoring capabilities through liquid biopsy enable treatment monitoring and resistance detection throughout the disease course [23,26].

### 4.2. Gene Expression Profiling (GEP)

Contemporary GEP assays have evolved significantly from early platforms, achieving sensitivity rates of 80–90% for TOO identification across expanded cancer type libraries [27,28,29]. These molecular classifiers analyze expression patterns of carefully selected gene panels to predict primary site with high confidence [30].

Improvements in GEP technology have substantially enhanced clinical utility. Enhanced accuracy has been achieved through improved algorithms and expanded reference libraries that better represent the molecular diversity of human cancers [20,27,28]. The integration of artificial intelligence and machine-learning approaches has improved pattern recognition capabilities, leading to more precise tissue-of-origin predictions [28,31,32,33,34]. Liquid biopsy compatibility has been developed, enabling circulating tumor DNA analysis for patients where tissue sampling is challenging [32]. Extensive clinical validation in CUP patient cohorts has provided robust evidence for the clinical utility of modern GEP platforms.

### 4.3. Circulating Tumor DNA (ctDNA) Methylation Classifiers

Emerging technologies such as the CUPiD classifier utilize DNA methylation patterns in ctDNA to predict tissue of origin [35]. These approaches offer several distinct advantages in the clinical management of CUP patients. The non-invasive sampling approach through blood-based analysis eliminates the need for repeat tissue biopsies and enables testing in patients with inaccessible lesions [35,36]. The high specificity achieved through methylation pattern analysis reflects the tissue-specific nature of epigenetic modifications. Real-time assessment capabilities allow for serial monitoring throughout the disease course, providing dynamic insights into tumor evolution [35]. Finally, these methylation-based classifiers serve as a complement to tissue-based assays, providing additional diagnostic information that can enhance overall diagnostic accuracy. Table 2 presents a synthesis of the molecular diagnostic platforms employed in CUP, including examples of their application in precision oncology [5].

## 5. Tumor-Agnostic Therapeutic Landscape

### 5.1. FDA-Approved Tumor-Agnostic Therapies

The approval of tissue-agnostic therapies has fundamentally altered the CUP treatment paradigm [24,37,38,39] (Table 3). Several classes of these agents have demonstrated clinical efficacy across multiple tumor types regardless of primary site of origin. Immunotherapy agents represent a major category of tumor-agnostic therapies with established efficacy in CUP patients. Pembrolizumab has received FDA approval for microsatellite instability-high (MSI-high) or mismatch repair deficient (dMMR) tumors as well as high tumor mutational burden (TMB-H) malignancies [40]. Dostarlimab has been approved specifically for dMMR solid tumors, while nivolumab has received approval for MSI-high/dMMR colorectal cancer.

Genomically targeted therapy agents have also shown remarkable efficacy in biomarker-selected populations. Larotrectinib, entrectinib and repotrectinib have been approved for NTRK fusion-positive tumors, representing the first truly tissue-agnostic targeted therapies. Selpercatinib has received approvals for RET fusion-positive solid tumors. The combination of dabrafenib plus trametinib has been approved for BRAF V600E-mutated solid tumors, extending the success of this combination beyond melanoma.

Several emerging agents represent the expanding landscape of tumor-agnostic therapies. Fam-trastuzumab deruxtecan has shown promising efficacy for HER2-positive solid tumors across multiple histologies and has been approved for all HER2-positive solid tumors defined by Immunohistochemistry 3 + (IHC3+), indicating that IHC is now emerging as a validated test in addition to NGS testing. Various PARP inhibitors are being investigated for homologous recombination deficient tumors, potentially expanding their utility beyond ovarian and breast cancers [41].

### 5.2. Clinical Implementation Challenges

Despite these therapeutic advances, several challenges limit the widespread application of tumor-agnostic therapies in CUP [42].The limited biomarker prevalence means that only a minority of CUP patients harbor currently actionable alterations, highlighting the need for continued biomarker discovery [43]. Access barriers persist, with geographic and economic disparities in molecular testing availability creating inequities in care delivery. Sequencing decisions regarding the optimal timing and sequencing of site-specific versus tumor-agnostic approaches remain uncertain, requiring evidence-based guidelines. Furthermore, resistance mechanisms to targeted therapies in the CUP context remain poorly understood, limiting the development of resistance-directed treatment strategies.

### 5.3. Research Challenges

All studies in CUP have inherent challenges. First, the limited prevalence of actionable biomarkers in cancer of unknown primary (CUP) restricts the proportion of patients who may benefit from targeted therapies. Second, access and reimbursement barriers, particularly in low-resource settings, pose significant challenges to the global implementation of precision oncology approaches. Third, heterogeneity in trial populations, including variability in diagnostic workup and prior treatments, may confound outcome comparisons and limit interpretability. Fourth, the potential for bias from single-center trials, especially those conducted at tertiary referral institutions, may affect the generalizability of findings. Fifth, short and variable follow-up durations across cohorts may underestimate long-term outcomes and late toxicities. Future multicenter studies with harmonized protocols are needed to validate and extend these observations.

## 6. Molecular Tumor Boards and Multidisciplinary Care

### 6.1. Role of Molecular Tumor Boards (MTBs)

MTBs have emerged as critical components of precision oncology infrastructure, facilitating multidisciplinary evaluation of genomic data and personalized treatment planning. In the CUP context, MTBs serve several essential functions that are particularly relevant given the complexity of these cases [44].

Complex case review represents a fundamental function of MTBs, involving the interpretation of molecular findings within the broader clinical context of each individual patient [44]. Treatment recommendations are developed through consensus-driven therapeutic guidance that incorporates multiple expert perspectives and current evidence. Clinical trial matching involves the systematic identification of appropriate investigational options for patients whose molecular profiles suggest potential benefit from experimental therapies. Finally, MTBs serve as educational platforms for knowledge dissemination and capacity building, ensuring that the broader oncology team remains current with rapidly evolving molecular diagnostics and targeted therapies [45].

### 6.2. Integration with Community Practice

Expanding MTB access to community oncology settings remains a critical implementation challenge [45]. Several potential solutions have been proposed to address this gap in precision oncology infrastructure. Virtual consultation platforms can provide remote access to MTB expertise, leveraging telemedicine technology to overcome geographic barriers. Decision support tools utilizing artificial intelligence can assist in the interpretation of molecular findings, providing evidence-based recommendations at the point of care [45,46]. Standardized protocols can offer simplified algorithms for common scenarios, enabling community providers to manage straightforward cases without specialist consultation. Educational initiatives, including comprehensive training programs for community providers, can build local expertise in molecular oncology and precision medicine principles [45].

## 7. Clinical Algorithm for Modern CUP Management

### Proposed Integrated Approach

**Figure 1:**
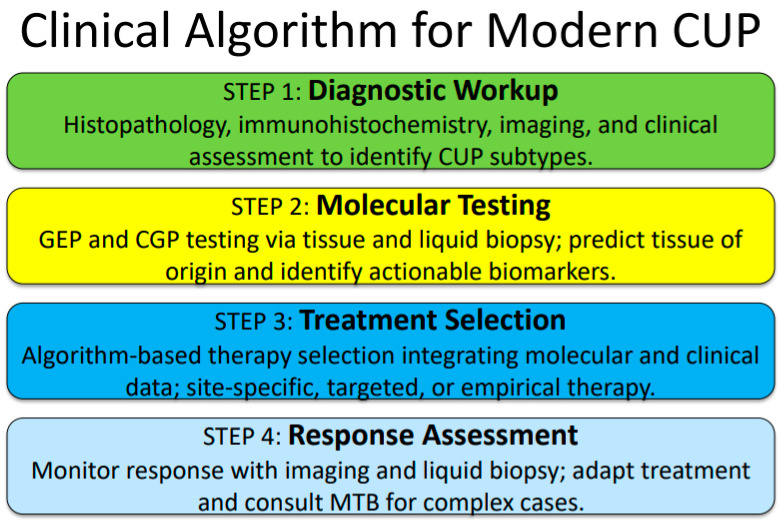
Clinical algorithm for modern CUP management.

Based on current evidence and expert consensus, we propose a comprehensive algorithm for CUP management that integrates molecular diagnostics with clinical decision-making. This approach consists of four sequential steps that build upon each other to optimize patient outcomes (Figure 1).

STEP 1 involves comprehensive diagnostic workup, beginning with standard histopathological evaluation complemented by appropriate immunohistochemistry panels. Baseline imaging and clinical assessment establish the extent of disease and performance status. This phase concludes with the identification of favorable versus unfavorable CUP subtypes based on established clinical and pathological criteria.

STEP 2 focuses on molecular testing strategy implementation, involving concurrent GEP and CGP testing using both tissue and liquid biopsy approaches when feasible. TOO prediction is performed with confidence scoring to guide subsequent treatment decisions. Simultaneously, actionable biomarker identification is conducted to determine eligibility for tumor-agnostic therapies.

STEP 3 encompasses treatment selection using an algorithm-based approach that integrates molecular findings with clinical factors. Patients with high-confidence tissue-of-origin predictions and actionable biomarkers receive a combined approach incorporating both site-specific and targeted therapies. Those with high-confidence tissue-of-origin predictions but no actionable biomarkers receive site-specific therapy based on the predicted primary site. Patients with indeterminate or low-confidence TOO predictions but actionable biomarkers receive tumor-agnostic therapy directed by the identified molecular alterations. Finally, patients without molecular guidance receive empirical chemotherapy with early reassessment for molecular testing if initial results are inconclusive.

STEP 4 involves response assessment and treatment adaptation, including regular clinical and radiological monitoring according to established guidelines. MTB consultation is recommended for cases with disease progression or complex clinical scenarios requiring multidisciplinary input.

## 8. Evidence Summary and Key Studies

### 8.1. Pivotal Clinical Trials Comparison

The evolution of CUP management can be understood through the lens of several pivotal clinical trials that have shaped current practice (Table 1). The Fudan CUP-001 phase III randomized controlled trial enrolled 182 patients and used progression-free survival as the primary endpoint [8]. The study demonstrated a hazard ratio of 0.64 (95% CI 0.46–0.89, *p* = 0.008), favoring GEP-guided site-specific therapy over empirical chemotherapy.

The CUPISCO phase II randomized controlled trial represented a larger effort with 573 patients, using progression-free survival as the primary endpoint [7]. This study achieved a hazard ratio of 0.69 (95% CI 0.52–0.91, *p* = 0.009), favoring CGP-guided targeted therapy over empirical chemotherapy [38].

In contrast, earlier studies demonstrated the limitations of molecular approaches when targeted therapies and immunotherapy were not available. The Hayashi et al. phase II randomized controlled trial with 130 patients showed a hazard ratio of 0.79 (95% CI 0.54–1.16, *p* = 0.23) for GEP-guided using microarray analysis SST, failing to reach statistical significance [12]. Similarly, the GEFCAPI 04 phase III trial with 243 patients demonstrated a hazard ratio of 0.87 (95% CI 0.57–1.33, *p* = 0.52), also failing to show significant benefit [11]. Considering the improved therapies for many advanced cancers today, these earlier studies are now outdated.

### 8.2. Biomarker Prevalence in CUP

Understanding the prevalence of actionable biomarkers in CUP is crucial for treatment planning and resource allocation. Microsatellite instability-high (MSI-H) or mismatch repair deficiency (dMMR) occurs in approximately 2–4% of CUP cases, with established therapeutic options including pembrolizumab and dostarlimab receiving FDA approval [6,24,47,48,49]. High tumor mutational burden (TMB-H) is found in 8–12% of cases, with pembrolizumab representing the primary FDA-approved therapeutic option [38]. NTRK fusions, while rare at less than 1% prevalence, have highly effective targeted therapies available, including larotrectinib, entrectinib and repotrectinib, which have received FDA approval. RET alterations occur in 1–2% of CUP cases, with selpercatinib providing an FDA-approved treatment option. BRAF V600E mutations are found in 2–5% of cases, with the combination of dabrafenib plus trametinib receiving FDA approval [39]. HER2 amplification occurs in 5–10% of cases, with trastuzumab deruxtecan showing promising results in investigational studies and having received approval for all IHC3+ cancers. Several biomarkers remain under investigation for CUP applications. Homologous recombination deficiency affects 10–15% of cases, with various PARP inhibitors under investigation for this indication. Although the identification and appreciation of actionable biomarkers is an essential element in CUP management, if a presumptive primary tumor is diagnosed, agnostic therapies may or may not be indicated as a first-line therapy depending on the specific cancer and the biomarker. The absence of actionable targets in many CUP cases remains a clinical challenge. As highlighted in the recent literature, molecular profiling may still provide diagnostic clarity or prognostic insights even when direct therapeutic targets are absent. Furthermore, the integration of transcriptomic and epigenetic data may help uncover cryptic lineage markers or indirect therapeutic vulnerabilities.

## 9. Perspectives and Clinical Considerations

The transition from empirical to molecularly guided therapy represents more than an incremental advance—it fundamentally challenges our conceptualization of CUP as a single-disease entity. Early prospective trials comparing site-specific therapy (SST) guided by molecular profiling versus empiric chemotherapy (EC) did not demonstrate clear superiority but had limitations such as heterogeneous patient populations, limited access to effective targeted agents, and evolving molecular platforms. However, more recent trials like Fudan CUP-001 have shown improved progression-free survival with SST guided by a 90-gene expression assay. The diagnostic approach must evolve to recognize that molecular characterization becomes as important as traditional pathological evaluation in determining optimal treatment strategies. Treatment selection increasingly relies on biomarker-driven decisions that may supersede anatomical considerations, fundamentally altering the decision-making process. Clinical trial design must adapt to emphasize basket trials and adaptive designs that become more relevant than traditional organ-specific studies [50]. Finally, regulatory considerations must evolve to accommodate tumor-agnostic approvals that gain precedence over site-specific indications.

## 10. Implementation Challenges and Solutions

Several key challenges must be addressed to successfully implement molecularly guided approaches in CUP management. Limited molecular testing access represents a significant barrier, requiring solutions that include expanded insurance coverage, development of point-of-care testing capabilities, and establishment of regional molecular pathology networks.

Interpretation complexity of molecular results poses another challenge, necessitating solutions such as standardized reporting formats, development of clinical decision support tools, and expanded access to MTB expertise. Treatment sequencing uncertainty creates clinical dilemmas that require evidence-based algorithms, comparative effectiveness studies, and the establishment of registry programs to guide optimal care.

Cost-effectiveness concerns must be addressed through comprehensive health economics studies that demonstrate value, development of risk stratification tools to optimize resource utilization, and optimization of testing strategies to balance comprehensiveness with cost efficiency.

## 11. Future Directions and Research Priorities

### 11.1. Technological Advances

The future of CUP management will be shaped by several emerging technological innovations. Artificial intelligence-enhanced diagnostics promise to revolutionize TOO prediction through sophisticated machine-learning algorithms that can identify complex patterns in molecular data. Multi-omics integration approaches will combine genomic, transcriptomic, and epigenomic profiling to provide unprecedented insights into tumor biology and therapeutic vulnerabilities.

Liquid biopsy evolution continues to advance with enhanced sensitivity for minimal residual disease detection, enabling more precise monitoring of treatment response and disease recurrence. Circulating tumor DNA (ctDNA) analysis and methylation classifiers such as CUPiD offer non-invasive, real-time diagnostic capabilities, which are particularly valuable when tissue samples are limited or exhausted by immunohistochemistry. Liquid biopsy also enables serial monitoring and complements tissue-based assays, enhancing diagnostic accuracy and therapeutic decision-making. Single-cell analysis technologies will facilitate tumor heterogeneity characterization and clonal evolution tracking, providing insights into resistance mechanisms and therapeutic adaptation.

### 11.2. Clinical Research and Care Delivery Priorities

Several key areas require focused research efforts to advance CUP management. Comparative effectiveness studies are needed to provide direct comparison of GEP-guided versus CGP-guided approaches, enabling evidence-based selection of optimal molecular strategies. Biomarker discovery efforts must continue to identify novel therapeutic targets in molecularly uncharacterized CUP cases, expanding treatment options for currently untreatable patients. Understanding resistance mechanisms to targeted therapies in the CUP context represents a critical knowledge gap that requires dedicated investigation. Quality-of-life studies examining patient-reported outcomes with molecular versus empirical approaches will inform treatment decision-making and healthcare policy. Innovation in healthcare delivery systems will be essential for the widespread implementation of precision oncology approaches in CUP. Telemedicine integration enables remote MTB consultations, overcoming geographic barriers to specialized expertise. Community implementation strategies must include enabling broader access to molecularly guided care. Global access initiatives through international collaborations can address disparities in molecular testing availability, particularly in resource-limited settings. Comprehensive training programs must develop educational curricula for molecular oncology in CUP, building workforce capacity to deliver precision medicine.

## 12. Conclusions and Future Outlook

The management of CUP stands at an inflection point. The convergence of advanced molecular diagnostics, tumor-agnostic therapies, and precision oncology principles has transformed CUP from a diagnosis of therapeutic nihilism to one of cautious optimism [51,52]. The landmark CUPISCO and Fudan CUP-001 trials have provided compelling evidence that molecularly guided approaches significantly improve outcomes compared to traditional empirical chemotherapy.

As we look toward the future, several key principles that will guide the continued evolution of CUP management emerge. A molecular-first approach represents the integration of GEP and CGP into routine CUP evaluation, signifying a paradigm shift from empirical chemotherapy and an organ-centric approach to a more biomarker-driven treatment selection. The recognition that complementary strategies involving GEP-guided site-specific therapy and CGP-guided tumor-agnostic approaches may be synergistic rather than competitive offers personalized treatment pathways based on individual molecular profiles.

Multidisciplinary integration through MTBs and precision oncology infrastructure is essential for optimal implementation of molecularly guided therapies. Continued innovation in artificial intelligence-enhanced diagnostics, multi-omics profiling, and liquid biopsy technologies promises further improvements in diagnostic accuracy and therapeutic targeting. Finally, ensuring equity and access to molecular testing and targeted therapies remains a critical challenge, requiring healthcare policy innovation and international collaboration. The journey from empirical chemotherapy to precision-based therapy in CUP exemplifies the broader transformation occurring throughout oncology. As molecular technologies continue to evolve and our understanding of cancer biology deepens, CUP may transition from an enigmatic syndrome to a molecularly defined collection of treatable diseases. This transformation requires sustained collaboration between clinicians, researchers, payers, and policymakers to ensure that every patient with CUP receives the benefit of molecularly informed precision care.

The paradigm shift in CUP management represents more than scientific progress— it embodies the promise of precision oncology to transform outcomes for patients facing previously intractable diagnoses. As we continue to unlock the molecular secrets of these enigmatic tumors, we move closer to a future where no cancer is truly “unknown” and every patient has access to personalized, biomarker-driven precision-based therapy.

## Figures and Tables

**Table 1:** Major clinical trials of CUP.

Study	Design	Population	Molecular Strategy	Primary Endpoint	Key Results	Clinical Impact
Fudan CUP-001 Liu et al. 2024 [8]	Phase III RCT N = 182	Unfavorable CUP First-line setting	90-gene GEP Site-specific therapy	Progression-free survival	PFS (9.6 vs. 6.6 months) hazard ratio of 0.68 (95% CI 0.50–0.94; *p* = 0.017).	Practice-changing NCCN/ ESMO impact
CUPISCO Kramer et al. 2024 [7]	Phase II RCT N = 573	Non-squamous unfavorable CUP Post-chemotherapy	CGP-based Tumor-agnostic therapy	Progression-free Survival	HR 0.72 (95% CI 0.56–0.92; *p* = 0.0079)	Tumor-agnostic validation Precision oncology
Hayashi et al. JCO 2019 [12]	Phase II RCT N = 150	CUP patients Treatment-naive	Microarray, GEP-guided Site-specific therapy	Overall Survival	HR 0.79 (95% CI 0.54–1.16) *p* = 0.23	Negative study Limited targeted options
GEFCAPI 04 Fizazi et al. 2019 [11]	Phase III RCT N = 150	Unfavorable CUP First-line setting	92-gene GEP Molecular profiling Tailored therapy	Overall Survival	HR 0.87 (95% CI 0.57–1.33) *p* = 0.52	Negative study Pre-precision era
Meta-Analysis Labaki et al. 2025 [13]	Systematic Review N = 1644	6 prospective studies 4 RCTs included	Various molecular approaches Pooled analysis	Overall Survival	Favors molecular therapy, Statistical significance	Confirmatory evidence Practice validation

NOTE: NCCN—National Comprehensive Cancer Network, ESMO—European Society for Medical Oncology, RCT—randomized controlled trial; CUP: Carcinoma of Unknown Primary; CUPISCO: Comprehensive profiling and molecularly guided therapy for carcinomas of unknown primary; JCO: Journal of Clinical Oncology; GEFCAPI: Groupe d’Etude Français des Carcinomes de Primatif Inconnu (French Study Group for Carcinoma of Unknown Primary); GEP: Gene Expression Profiling; CGP: Comprehensive Genomic Profiling; PFS: Progression-Free Survival; CI: Confidence Interval; HR: Hazard Ratio.

**Table 2:** Molecular Diagnostic Technologies employed in CUP.

Platform Type	Technology	Sample Type	Key Features	Accuracy	Clinical Utility	Limitations
Gene Expression Profiling (GEP)	90-and 92-gene assays RT-PCR/NGS	FFPE tissue Fresh tissue	TOO prediction 50+ cancer types/subtypes Confidence scoring	80–90% sensitivity 85–95% specificity	Site-specific therapy guidance Treatment selection	Reference library gaps Overlapping diagnoses Sample quality dependent
Comprehensive Genomic Profiling (CGP)	300+ gene NGS WES/WGS	Tissue biopsy Liquid biopsy	Actionable mutations MSI/TMB status Gene fusions CNV analysis	95%+ analytical accuracy Variable actionability	Tumor-agnostic therapy Clinical trial matching	Cost considerations Interpretation complexity Limited actionable targets
cfDNA Methylation	CUPiD classifier Methylation arrays	Plasma/serum Blood-based	Non-invasive Real-time monitoring TOO classification	High tissue specificity Good sensitivity	Serial monitoring Minimal invasive diagnosis	Technology emerging Limited validation Cost barriers
Multi-omics Integration	AI/ML algorithms Combined platforms	Multi-modal Tissue + liquid	Enhanced accuracy Pattern recognition Integrated reporting	Potentially superior Under development	Future precision medicine Comprehensive profiling	Developmental stage Regulatory pathway Infrastructure needs

Note: TOO—Tissue of Origin, RT-PCR—Reverse Transcription Polymerase Chain Reaction. FFPE—Formalin-Fixed, Paraffin-Embedded, WES—Whole-Exome Sequencing, WGS—Whole-Genome Sequencing; NGS: Next-Generation Sequencing; CUPiD: Cancer of Unknown Primary integrated Diagnostics; AI/ML: Artificial Intelligence/Machine Learning; MSI/TMB: Microsatellite Instability/Tumor Mutational Burden; CNV: Copy Number Variation.

**Table 3:** Tissue-agnostic drugs that could be deployed in treatment of CUP.

Agent(s)	Target/Biomarker	Mechanism	Prevalence in CUP	Approval Status	Key Efficacy Data	Clinical Considerations
Pembrolizumab	MSI-H/dMMR TMB-H (≥10 mut/Mb)	PD-1 inhibitor Immune checkpoint	2–4% MSI-H 8–12% TMB-H	FDA-Ap	ORR 29–57% Durable responses OS benefit	First-line option Biomarker testing essential irAE monitoring
Larotrectinib Entrectinib Repotrectinib	NTRK gene fusions TRK A/B/C	TRK inhibitor ATP-competitive	<1% prevalence	FDA-Ap	ORR 75–80% Tumor-agnostic efficacy CNS activity	Rare but actionable Resistance mechanisms
Selpercatinib	RET alterations Fusions/mutations	Selective RET inhibitor Multi-kinase activity	1–2% prevalence	FDA-Ap	ORR 60–85% CNS penetration Durable responses	Tissue-agnostic approval Multiple RET alterations
Dabrafenib + Trametinib	BRAF V600E V600K mutations	BRAF + MEK inhibition MAPK pathway	2–5% prevalence	FDA-Ap	ORR 46% 6-month PFS 46% Multiple histologies	Combination required
Trastuzumab Deruxtecan	HER2 overexpression IHC 3+/ISH+	ADC technology Topoisomerase I inhibitor	5–10% prevalence	FDA-ap	ORR 37–54% Multiple solid tumors Low HER2 activity	ILD monitoring Bystander effect

Note: RAE—Immune-Related Adverse Event, ILD—Interstitial Lung Disease, ISH—in situ Hybridization, IHC—Immunohistochemistry, ORR—Overall Response Rate, OS—Overall Survival; CUP: Cancer of Unknown Primary; MSI-H: Microsatellite Instability-High; dMMR: Deficient Mismatch Repair; TMB-H: Tumor Mutational Burden-High; NTRK: Neurotrophic Tyrosine Receptor Kinase (gene); TRK: Tropomyosin Receptor Kinase (protein); RET: REarranged during Transfection (proto-oncogene); BRAF: B-Rapidly Accelerated Fibrosarcoma (proto-oncogene); HER2: Human Epidermal Growth Factor Receptor 2; PD: Progressive Disease; ATP: Adenosine Triphosphate; MEK: Mitogen-Activated Protein Kinase Kinase; MAPK: Mitogen-Activated Protein Kinase; ADC: Antibody-Drug Conjugate; FDA-Ap: FDA-Approved; ORR: Overall Response Rate; OS: Overall Survival; CNS: Central Nervous System; irAE: Immune-Related Adverse Events.

## Data Availability

No new data were created or analyzed in this study. Data sharing is not applicable to this article.
